# Persistence of *Yersinia pestis* in Soil Under Natural Conditions

**DOI:** 10.3201/eid1406.080029

**Published:** 2008-06

**Authors:** Rebecca J. Eisen, Jeannine M. Petersen, Charles L. Higgins, David Wong, Craig E. Levy, Paul S. Mead, Martin E. Schriefer, Kevin S. Griffith, Kenneth L. Gage, C. Ben Beard

**Affiliations:** * Centers for Disease Control and Prevention, Fort Collins, Colorado, USA; † National Park Service, Washington, DC, USA; ‡ Arizona Department of Health Services, Phoenix, Arizona, USA

**Keywords:** Yersinia pestis, soil, plague, dispatch

## Abstract

As part of a fatal human plague case investigation, we showed that the plague bacterium, *Yersinia pestis,* can survive for at least 24 days in contaminated soil under natural conditions. These results have implications for defining plague foci, persistence, transmission, and bioremediation after a natural or intentional exposure to *Y. pestis*.

Plague is a rare, but highly virulent, zoonotic disease characterized by quiescent and epizootic periods (*1*). Although the etiologic agent, *Yersinia pestis*, can be transmitted through direct contact with an infectious source or inhalation of infectious respiratory droplets, flea-borne transmission is the most common mechanism of exposure (*1*). Most human cases are believed to occur during epizootic periods when highly susceptible hosts die in large numbers and their fleas are forced to parasitize hosts upon which they would not ordinarily feed, including humans (*2*). Despite over a century of research, we lack a clear understanding of how *Y. pestis* is able to rapidly disseminate in host populations during epizootics or how it persists during interepizootic periods (*2*–*6*). What limits the geographic distribution of the organism is also unclear. For example, why is the plague bacterium endemic west of the 100th meridian in the United States, but not in eastern states despite several known introductions (*7*)?

Persistence of *Y. pestis* in soil has been suggested as a possible mechanism of interepizootic persistence, epizootic spread, and as a factor defining plague foci (*2*,*3*,*5*,*7*,*8*). Although *Y. pestis* recently evolved from an enteric bacterium, *Y. pseudotuberuclosis*, that can survive for long periods in soil and water, studies have shown that selection for vector-borne transmission has resulted in the loss of many of these survival mechanisms. This suggests that long-term persistence outside of the host or vector is unlikely (*9*–*11*). Previous studies have demonstrated survival of *Y. pestis* in soil under artificial conditions (*2*,*3*,*12*–*14*). However, survival of *Y. pestis* in soil under natural exposure conditions has not been examined in North America.

## The Study

As part of an environmental investigation of a fatal human plague case in Grand Canyon National Park, Arizona, in 2007, we tested the viability of *Y. pestis* in naturally contaminated soil. The case-patient, a wildlife biologist, was infected through direct contact with a mountain lion carcass, which was subsequently confirmed to be positive for *Y. pestis* based on direct fluorescent antibody (DFA) testing (which targets the *Y. pestis*–specific F1 antigen), culture isolation, and lysis with a *Y. pestis* temperature-specific bacteriophage (*15*). The animal was wearing a radio collar, and we determined the date of its death (October 26, 2007) on the basis of its lack of movement. The case-patient had recorded the location at which he encountered the carcass and had taken photographs of the remains, which showed a large pool of blood in the soil under the animal’s mouth and nose. During our field investigation, ≈3 weeks after the mountain lion’s death, we used global positioning satellite coordinates and photographs to identify the exact location of the blood-contaminated soil. We collected ≈200 mL of soil from this location at depths of up to ≈15 cm from the surface.

After collection, the soil was shipped for analysis to the Bacterial Diseases Branch of the Centers for Disease Control and Prevention in Fort Collins, Colorado. Four soil samples of ≈5 mL each were suspended in a total volume of 20 mL of sterile physiologic saline (0.85% NaCl). Samples were vortexed briefly and allowed to settle for ≈2 min before aliquots of 0.5 mL were drawn into individual syringes and injected subcutaneously into 4 Swiss-Webster strain mice (ACUC Protocol 00–06–018-MUS). Within 12 hours of inoculation, 1 mouse became moribund, and liver and spleen samples were cultured on cefsulodin-Irgasan-novobiocin agar. Colonies consistent with *Y. pestis* morphology were subcultured on sheep blood agar. A DFA test of this isolate was positive, demonstrating the presence of F1 antigen, which is unique to *Y. pestis.* The isolate was confirmed as *Y. pestis* by lysis with a *Y. pestis* temperature–specific bacteriophage (*15*). Additionally, the isolate was urease negative. Biotyping (glycerol fermentation and nitrate reduction) of the soil and mountain lion isolates indicated biovar *orientalis*.

Of the 3 remaining mice, 1 became moribund after 7 days and was euthanized; 2 did not become moribund and were euthanized 21 days postexposure. Culture of the necropsied tissues yielded no additional isolates of *Y. pestis*. Pulsed-field gel electrophoresis (PFGE) typing with *Asc*I was performed with the soil isolate, the isolate recovered from the mountain lion, and the isolate obtained from the case-patient (*16*). The PFGE patterns were indistinguishable, showing that the *Y. pestis* in the soil originated through contamination by this animal (Figure). Although direct plating of the soil followed by quantification of CFU would have been useful for assessing the abundance of *Y. pestis* in the soil, this was not possible because numerous contaminants were present in the soil.

**Figure:**
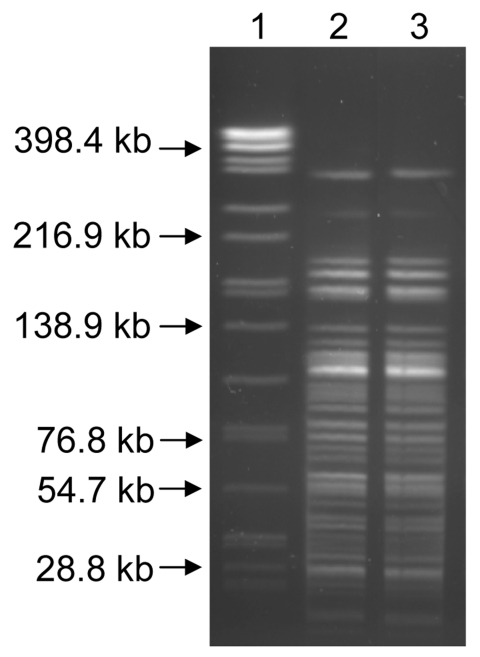
*Asc*I pulsed-field gel electrophoresis patterns for the *Yersinia pestis* isolates recovered from soil (lane 3) and the mountain lion (lane 2). Lane 1, *Salmonella enterica* serotype Braenderup standard.

## Conclusions

It is unclear by what mechanism *Y. pestis* was able to persist in the soil. Perhaps the infected animal’s blood created a nutrient-enriched environment in which the bacteria could survive. Alternatively, adherence to soil invertebrates may have prolonged bacterial viability (*17*). The contamination occurred within a protected rock outcrop that had limited exposure to UV light and during late October, when ambient temperatures were low. These microclimatic conditions, which are similar to those of burrows used by epizootic hosts such as prairie dogs, could have contributed to survival of the bacteria.

These results are preliminary and do not address 1) the maximum time that plague bacteria can persist in soil under natural conditions, 2) possible mechanisms by which the bacteria are able to persist in the soil, or 3) whether the contaminated soil is infectious to susceptible hosts that might come into contact with the soil. Answers to these questions might shed light on the intriguing, long-standing mysteries of how *Y. pestis* persists during interepizootic periods and whether soil type could limit its geographic distribution. From a public health or bioterrorism preparedness perspective, answers to these questions are necessary for evidence-based recommendations on bioremediation after natural or intentional contamination of soil by *Y. pestis*. Previous studies evaluating viability of *Y. pestis* on manufactured surfaces (e.g., steel, glass) have shown that survival is typically <72 hours (*18*). Our data emphasize the need to reevaluate the duration of persistence in soil and other natural media.
